# Individual differences in motives, preferences, and pathology in video games: the gaming attitudes, motives, and experiences scales (GAMES)

**DOI:** 10.3389/fpsyg.2013.00608

**Published:** 2013-09-09

**Authors:** Joseph Hilgard, Christopher R. Engelhardt, Bruce D. Bartholow

**Affiliations:** Social-Cognitive Neuroscience Laboratory, Department of Psychology, University of Missouri-ColumbiaColumbia, MO, USA

**Keywords:** video games, game pathology, game addiction, motives for game play, player personality

## Abstract

A new measure of individual habits and preferences in video game use is developed in order to better study the risk factors of pathological game use (i.e., excessively frequent or prolonged use, sometimes called “game addiction”). This measure was distributed to internet message boards for game enthusiasts and to college undergraduates. An exploratory factor analysis identified 9 factors: Story, Violent Catharsis, Violent Reward, Social Interaction, Escapism, Loss-Sensitivity, Customization, Grinding, and Autonomy. These factors demonstrated excellent fit in a subsequent confirmatory factor analysis, and, importantly, were found to reliably discriminate between inter-individual game preferences (e.g., *Super Mario Brothers* as compared to *Call of Duty*). Moreover, three factors were significantly related to pathological game use: the use of games to escape daily life, the use of games as a social outlet, and positive attitudes toward the steady accumulation of in-game rewards. The current research identifies individual preferences and motives relevant to understanding video game players' evaluations of different games and risk factors for pathological video game use.

## Introduction

The video game industry is among the fastest growing sectors of the US economy. This industry generated $25 billion in sales in 2011, and from 2005 to 2009, boasted an annual growth rate of more than 5 times the growth rate of the entire US economy over that same period (Siwek, 2010). Evidence correspondingly suggests that individuals play video games now more than ever (see Anderson et al., 2007). For example, Gentile (2009) reported that individuals aged 8 to 18 play video games for nearly 15 h per week on average. Whereas considerable research has focused on the effects of game contents, particularly violence (see Anderson et al., 2010), comparatively little research has investigated factors that might contribute to pathological (sometimes called “addictive”) patterns of video game play (Fisher, 1994; Chiu et al., 2004; Charlton and Danforth, 2007; Gentile, 2009; Gentile et al., 2011) and what motivates people to play and prefer certain games over others (Przybylski et al., 2010). The current report contributes to the emerging literature on individual differences in game preferences and motives by developing and validating an instrument to measure these constructs.

As video games have grown in universal popularity, they have also grown in diversity. Today's video games constitute a myriad of different studios, developers, and genres. Specific games often contain a variety of options, allowing the player to interact with the game in many different ways. With so many options both between and within video games, it is not surprising that individuals often prefer one game type to another, much like with other forms of popular media. Just as one movie enthusiast may favor Spielberg as another prefers Tarantino, so too might one gamer enjoy Sid Meier's strategy games (e.g., *Civilization*) while another prefers Ken Levine's narrative-based first-person shooters (e.g., *Bioshock*).

The intuitive idea that different gamers have different motives and preferences for video games is supported by recent research. For example, Ryan et al. (2006) examined player motivations through the application of self-determination theory (SDT) (Deci and Ryan, 1985). SDT predicts that players should enjoy a video game insofar as it satisfies a player's basic psychological need for autonomy (a sense of control), competence (a sense that one is performing well), and relatedness (friends and relationships). Consistent with this hypothesis, Ryan et al. (2006) found that, across all players, the subjective experience of autonomy, competence, and relatedness during play made games more motivating and appealing to the player. Furthermore, critically well-reviewed games (e.g., *The Legend of Zelda: Ocarina of Time*) tended to better satisfy needs than did critical flops (e.g., *A Bug's Life*). Importantly, different players found the same critically successful games to be differently satisfying of their SDT needs and thereby differently enjoyable. This phenomenon suggests that individual differences in player preferences may moderate whether a particular game satisfies or stifles SDT needs, and thus, which players will enjoy which games.

Researchers, players and game developers alike have long been interested in measuring individual differences in game motivations and preferences. Theories of “player personality” began with Bartle (1996), who speculated that players are separated into one of four types according to the degree to which each player prefers to act upon (as opposed to interact with) the game world and the degree to which each player enjoys interaction with other players. More recently, Sherry et al. (2006) interviewed American undergraduate students in focus groups to determine dimensions of video game use motivations. They identified six dimensions: Arousal, Challenge, Competition, Diversion, Fantasy, and Social Interaction. These dimensions were found to be strong predictors of video game play in a subsequent survey, such that higher ratings of Arousal, Diversion, and Social Interaction were associated with more hours of weekly video game use.

Another model comes from Yee et al. (2012; Yee, 2006a,b). Yee and colleagues surveyed players of Massively Multiplayer Online Role-Playing Games (MMORPGs), identifying three factors composed of ten subcomponents. These factors included: Achievement, consisting of subcomponents Advancement, Mechanics, and Competition; Social, consisting of Socializing, Relationships, and Teamwork; and Immersion, consisting of Discovery, Role-Playing, Customization, and Escapism. These factors have since been found to relate to players' domains of greatest advancement in *World of Warcraft*. For example, more Achievement-oriented players had a greater proportion of in-game “achievements” in player-vs.-player combat and cooperative dungeon raids, whereas more Immersion-oriented players had proportionately more achievements related to exploration (Yee et al., 2012).

These three approaches each have attempted to measure and explain individual preferences in games—an approach that could explain how the same game satisfies one player's SDT needs while stifling another's. Indeed, Yee's (2006a) factors seem particularly in line with SDT motives: players differ to the extent that they use games to fulfill feelings of Competency (i.e., Yee's Achievement factor) or Relatedness (i.e., Yee's Social factor). Sherry et al. (2006) similarly measured attitudes toward competition and social interaction. Variance in these measures would seem to indicate that players vary in the SDT needs they seek to fulfill through game use. For example, one player may use games to experience Relatedness, while another instead uses them to experience Competency.

Since motive models can predict hours spent playing video games (e.g., Sherry et al., 2006; Yee, 2006b), understanding individual differences in game motives may be crucial to understanding factors that lead to problematic video game use, or what some have called “pathological video game use” (Gentile, 2009) or “pathological technology use” (Gentile et al., 2013). Originally adapted from the Diagnostic and Statistical Manual, fourth edition (DSM-IV; American Psychiatric Association, 2000) criteria for gambling addiction, measures of pathological video game use have steadily improved in reliability and validity. Like gambling, excessive use of video games can have numerous adverse consequences for the individual. For example, pathological video game use is associated with depression, anxiety, social phobia, and impaired school performance (Gentile et al., 2011). In one extreme case, a woman became so preoccupied with the game *World of Warcraft* that her 3-year-old daughter died of neglect (Las Cruces Sun-News; Meeks, 2011).

Individual differences in players' motives and preferences in video game use may determine which gamers enjoy healthy, balanced game use and which gamers are at risk for pathological game use. For example, much like how coping motives are associated with alcohol abuse (Cooper et al., 1988, 1992, 1995), evidence correspondingly suggests that players who use video games to escape from their problems experience greater problems because of their game use (Yee, 2006b; Kneer and Glock, 2013).

Indeed, there is reason to believe that preferences for certain game features may be related to pathological game use. Researchers have suggested that certain features make some games more addictive than others (Wan and Chiou, 2007; King and Delfabbro, 2009; King et al., 2011). For instance, internet games containing social interaction are often found to be more addictive than offline, single player video games (Thomas and Martin, 2010). Also, addicts, as compared to controls, report greater enjoyment in finding rare in-game items that can help their game character “rank up” (King et al., 2010). For these reasons, researchers have tended to suspect Massively Multiplayer Online Role-Playing Games (MMORPGs) as being particularly addictive (Linderoth and Bennerstedt, 2007; Hellström et al., 2012; Kneer and Glock, 2013), sometimes to the extent of excluding all other genres from studies of game pathology (e.g., Yee, 2006b; Hellström et al., 2012). By identifying differences in game preferences, it may be possible to identify players who prefer certain game styles and to determine whether they experience more symptoms of pathological game use than other players. For example, players may vary in the extent to which they are motivated by in-game items or online social interaction. Players who are particularly excited by in-game rewards, such as time-consuming achievements and rare items, may find themselves compelled to play for excessive periods. Similarly, players who prefer games with a strong social component may find themselves more likely to become obligated to play the game, possibly leading to conflicts between game life and real life.

In-game behavior reinforcement is potentially related to pathological game use. A number of studies have linked video game play to the activation of reward networks also involved in drug use and addiction (Koepp et al., 1998; Hoeft et al., 2008). Game designers looking to keep players engaged are now applying principles of operant conditioning to game design (Skinner et al., 1997; Hopson, 2001). In-game rewards are often dispensed according to a variable-ratio reward schedule, in which a variable number of actions are required to earn a reward. For example, a *Diablo* player may find a powerful weapon on the very next monster she slays, or that weapon may not be found until a thousand monsters later. This reward schedule fosters rapid, frequent engagement of behavior, and the learned behavior is slow to extinguish in the absence of reward. The structure and importance of these reward schedules vary between games, which may cause certain kinds of video game to be more closely related to pathology. Supporting this hypothesis, Yee (2006b) found that players more motivated by the prospect of completing goals and accumulating rare items exhibited more symptoms of pathological game use. While most SDT perspectives have focused on skill-based challenge as a source of fulfillment of competency needs (Przybylski et al., 2010), the accumulation of rewards also can lead to a powerful player avatar and feelings of achievement and progress, likely satisfying SDT competency needs, even in the absence of challenge.

Social obligation may be yet another hazardous game feature. In many online games, players must work together to achieve higher-order goals. In the case that a player is an essential member of a group, the player is socially obligated to play for as long as the rest of the group wants to play (King and Delfabbro, 2009). “Social games” such as Farmville also strive to make players obligated to play at regular intervals by making players depend on one another for daily allotments of in-game resources. Despite these potentially time-consuming social obligations, many players enjoy multiplayer games, probably because the features associated with social games provide opportunities for players to fulfill their SDT relatedness needs.

Measurements of player motives stand to inform which players are particularly motivated by the above game features, and thus whether these game features are associated with greater pathology. However, there are a number of ways in which measurements of player motives could be improved to better understand preferences and pathology. First, since the development of other game motives and preferences measures (Sherry et al., 2006; Yee, 2006a), stories have emerged as a major motivation in video game use, with some players likening their experience to more traditional media forms like movies, books, or art. Next, previous efforts have been restricted to small subsets of the gaming population. For example, Yee (2006a,b, 2012) studied only players of MMORPGs, a single genre of video game, while Sherry et al.'s research (2006) focused on gamers of age 23 and younger.

Importantly, no measure to date has demonstrated an ability to discriminate between players of different games. The ability to discriminate between game platforms, genres, and titles is tantamount to understanding the differences between diverse video games and their different potentials to inspire pathological use. Thus, a comprehensive and externally valid measure of preferences should be able to discriminate between fans of different styles of game and even different gaming platforms. For example, people who play games primarily through incidental platforms such as Facebook or iPhone (sometimes called “casual gamers”) should be measurably different from people who purchase game consoles specifically to play video games. Similarly, fans of different games should vary in their enthusiasm for different game features. For example, some video games have been critically praised for their storytelling (e.g., *Mass Effect, Bioshock*) (Dahlen, 2007; Villoria, 2010), whereas in other games, story is an incidental framing device, sometimes ignored outright (e.g., *Super Mario Brothers, Team Fortress 2, DOOM*). Similarly, some games have lively multiplayer communities (e.g., *Minecraft, World of Warcraft*) while others are exclusively single-player experiences. Some games allow for the steady accumulation of level-ups and items over time (e.g., *Skyrim*, *World of Warcraft, Call of Duty*), while other games take place in isolated, non-cumulative games (e.g., *Starcraft, Civilization, Tetris*). We expect that players may have preferences for one set of game mechanisms over another, creating meaningful and predictable patterns of covariation between favorite game franchises and motive measurements.

The purpose of the current study was to investigate video game preferences and motives among a broad sample of participants, and in doing so, to develop and provide initial validity information on a new measure of these constructs. This effort improves upon previous work by attempting to measure a wider variety of potential motives and by studying a more diverse population of gamers, including players of numerous game genres and infrequent (casual) players. By validating this measure through comparisons to preferred video games and gaming platforms, this study is able to explore whether certain game motives, genres of game, or game platforms are associated with greater incidence of pathological game use.

## Methods

### Participants

Participants were collected from two sources. First, Internet volunteers were recruited, with moderator permission, through forum postings at www.reddit.com/r/truegaming, www.reddit.com/r/girlgamers, forums.penny-arcade.com, www.rpgcodex.net, www.minecraftforum.net/forum, www.skyrimforums.org, www.conquerorworm.net, and www.badgame.net. Forum posters had the advantage of being plentiful and were willing to volunteer their time for minimal compensation, but self-selection pressures caused these participants to be overwhelmingly male gamers who played daily. Thus, the survey was also distributed to college undergraduates, advertised as a “survey of having fun” rather than a “video game survey,” in order to sample from more females and less frequent players.

The current sample included 1689 individuals recruited from internet discussion forums who completed the survey for a chance to win one of ten $20 Amazon gift cards. (87% male, 79% Non-Hispanic White, 4% Asian, 1% Indian, 1% Arab, 2% Native American, 4% Hispanic White, and 7% not otherwise specified. The average age was 23.4, *SD* = 6.03, range = 10–66.) An additional 300 college undergraduates were recruited from the University of Missouri, who completed the survey in exchange for partial course credit. (27% male, 82% Non-Hispanic White, 2% Hispanic White, 8% Black, 2% Non-Hispanic Asian, 1% Hispanic Asian, and 3% not otherwise specified. Their average age was 18.4, *SD* = 1.21, range = 17–34).

The survey was conducted through www.qualtrics.com. The research was approved by the University of Missouri-Columbia IRB, and informed consent was obtained from all subjects.

### Measures

#### Demographic information

Participants indicated their age, sex, race (“*White*,” Black, Asian, Arabic, Indian, Native American, Other), and ethnicity (“*Hispanic*” or “*Not Hispanic*”).

#### Video game exposure

Participants indicated how casual they were about playing video games on a scale ranging from 1 (*Very hardcore*) to 5 (*Very casual*) and how frequently they played such games (*Daily, 2–3 times/week, weekly, 2–3 times/month, monthly, less than monthly, never*). Participants also indicated how many hours (on weekdays and weekends) they spent playing video games during the following 6 h intervals: midnight to 6 AM, 6 AM to noon, noon to 6 PM, and 6 PM to midnight. They also indicated what proportion of their spare time was spent playing video games on a scale ranging from 1 (*Almost none of my spare time*) to 5 (*Almost all of my spare time*).

#### Preferred games

Participants were also asked to list, via open response, three of their favorite games (including non-video games) and three games that they were currently playing.

To increase statistical power, this item collapsed across games within franchises when individual games were reasonably similar. For example, different entries within the *Final Fantasy* franchise were collapsed together, with the exception of *Final Fantasy XI* and *Final Fantasy XIV*, which were massively multiplayer online games instead of single-player Japanese role-playing games. Similarly, the 1990s turn-based role-playing games *Fallout 1* and *Fallout 2* were combined to a single entry, while the 2008 first-person shooter role-playing game *Fallout 3* was kept as its own separate entry. The *World of Warcraft* MMO was kept separate from the *Warcraft* real-time strategy franchise. Since each successive *The Elder Scrolls* game has had equally fervent fans and detractors, *Morrowind*, *Oblivion*, and *Skyrim* were each kept as separate entries.

Responses were restricted to the twenty most frequently-indicated favorite games. These included: *The Legend of Zelda* franchise, *Final Fantasy* franchise (excluding MMOs), *Half-Life* franchise, *Mass Effect* franchise, *Fallout* 1 and 2, *Deus Ex* 1, *Super Mario* franchise (excluding spinoffs like *Mario Party* or *Mario Kart*), *Portal* franchise, *Skyrim*, *Halo* franchise (excluding the spinoff *Halo Wars*), *Planescape: Torment*, *Pokemon* franchise, *Call of Duty* franchise, *Morrowind*, *Team Fortress 2*, *Minecraft*, *Grand Theft Auto* franchise, *World of Warcraft*, *Baldur's Gate 2*, and *Bioshock* franchise.

Additionally, participants indicated via checklist which media platforms they *most typically* use to play games (PC, Nintendo Wii, Sony Playstation 3, Microsoft XBOX 360, Nintendo DS, Sony Playstation Portable, cellular phone, Facebook, board or card games, pen and paper roleplaying, real-life sports, arcade cabinets, and other).

#### Gaming attitudes, motives, and experiences scales

Participants answered 121 video game related questions intended to assess their motives and preferences for such media. Of these items, 20 were taken from the Video Game Uses and Gratifications Instrument developed by Sherry et al. (2006) (e.g., “I play video games because they excite me.”). This six-factor scale models individual differences in game uses and gratifications as a function of Competition (α = 0.86), Challenge (α = 0.80), Social Interaction (α = 0.81), Diversion (α = 0.89), Fantasy (α = 0.88), and Arousal (α = 0.85). An additional 100 items were developed by the experimenters to measure other possible individual differences in game preferences and motives. Hypothesized preferences and motives included emotion regulation, transportation, ability to enjoy a loss, customization, catharsis, and violence, among others. Items were answered using a 5-point Likert scale ranging from 1 (*Strongly disagree*) to 5 (*Strongly agree)* (e.g., “I find easy games to be too boring” or “I prefer games that make me rely on my teammates.”). Participants were given a “Not applicable” response option in the case that they had no experience with an item. Items were presented in a random order across participants. One survey item requested that participants indicate a “*Neither Agree nor Disagree*” response. This item served as a proxy for attention. Subjects who failed to mark this item appropriately were excluded.

#### Video game pathology

After completing the motives and preferences survey, participants completed a measure of pathological video game use developed by Gentile (2009). Participants were asked if they had experienced each of 15 symptoms of pathological video game use. For example, the questionnaire asks whether participants experience withdrawal (“In the past year, have you become restless or irritable when attempting to cut down or stop playing video games?”), conflict with work (“In the past year, have you skipped classes or work in order to play video games?”), and conflict with others (“In the past year, have you ever lied to family or friends about how much you play video games?”). Participants indicated whether they had experienced each symptom by responding “*Yes*,” “*No*,” “*Sometimes*,” or “*Not Applicable*.” “Sometimes” responses were considered equivalent to half a “yes” response (yes = 1, sometimes = 0.5, no or N/A = 0), as this approach yielded the greatest reliability in previous research (α = 0.78) (Gentile et al., under review).

## Results

### Samples

Compared to the internet sample, the undergraduate sample was younger [Welch's *t*_(1598)_ = 27.42, *p* < 0.001], proportionately more female (87 vs. 27%, *G* = 414, 1 d.f., *p* < 0.001), more casual about video games [Welch's *t*_(365)_ = 26.33, *p* < 0.001], played less frequently [Welch's *t*_(303)_ = 20.59, *p* < 0.001], and spent a smaller proportion of their spare time on video games [Welch's *t*_(403)_ = 30.62, *p* < 0.001]. The undergraduate sample thus adds diversity to the study sample, making the following analyses better representative of game use in general rather than game use only by serious players.

A large number of participants from the initial sample (*N* = 1280) were eliminated from the final sample due to missing data (e.g., “clicking through” the online survey without responding to most items; starting the survey and not finishing it) or responding “Not Applicable” to some items. We also removed participants who did not respond with “3” to our attention item (*N* =27), and removed participants who responded “3” to every item on the survey (*N* = 3). Participants with a Mahalonobis distance three standard deviations above the mean were discarded as multivariate outliers (*N* = 7), leaving 672 subjects for this stage of analysis (Mean age = 22.6 (5.51), 79% male, 85% Non-Hispanic White, 4% Hispanic White, 2% Black, 5% Asian, 1% Indian, 1% Arab, 2% Native American, 5% not otherwise specified).

### Factor structure

Many items were highly skewed. In order to improve the performance of the factor analysis, we recoded rare and extreme responses to the next-most extreme response (see Wilcox, 1995). For example, on an item where only three participants responded “5—Strongly Agree,” that response was recoded as a “4—Agree.” Forty-five of the 121 items were adjusted in this manner^1^.

To establish and validate our motives and preferences factor structure, we conducted a split-halves exploratory factor analysis (EFA) and confirmatory factor analysis (CFA) process. Participants were randomly assigned to the EFA or CFA group. Of the 332 participants assigned to the EFA group, 50 were college undergraduates.

EFA was performed in an iterative process using the “nFactors” package for R (Raiche and Magis, 2010). First, the data were submitted to a parallel analysis (see Fabrigar et al., 1999). Parallel analysis performs a principal factor decomposition of the data matrix and compares it to a principal factor decomposition of a randomized data matrix. This analysis yields components whose eigenvalues (magnitudes) are greater in the observed data relative to the randomized data. Next, data were submitted to an EFA using an oblique promax rotation with the recommended number of factors from the parallel analysis extracted from the original data matrix. We inspected the factor loadings and dropped items with weak loadings (no loading > 0.30). We also dropped items that demonstrated complex loadings (items that loaded > 0.30 on more than one factor) for two consecutive iterations. We repeated this iterative process (parallel analysis and then dropping poor and complex items) until a stable solution was reached (i.e., no items reached criteria for exclusion).

The final solution consisted of nine factors. A tenth factor, Procrastination, was recommended by parallel analysis, but it was composed of only two items with very similar wordings and was discarded. Two items from Sherry et al. (2006) failed to load upon their previously validated factor: “I find it very rewarding to get to the next level” and “I play until I win a game or complete a level” loaded upon the Grinding factor rather than any challenge-related factor. This was likely due to the ambiguity of the word “level,” which can apply either to a portion or stage of a game (e.g., in an action game, beating a level and moving on to the next one) or to the accumulation of avatar strength (e.g., “leveling up” in a role-playing game, thereby becoming stronger). Thus, although the loadings of these items were approximately simple, the items were discarded to avoid any ambiguity.

A complete list of the hypothetical factor names and meanings may be seen in Table 1. The items that remained after the final iteration are listed in Table 2, sorted by factor and renumbered. Factor loadings for these items are available in Table 3. Inter-factor correlations and Cronbach's alphas for each factor are summarized in Table 4.

**Table 1:** **GAMES factors and their hypothesized meanings**.

**Factor**	**Meaning**
Story	Whether game stories are important, engaging, and emotionally compelling.
Violent catharsis	Whether game violence is perceived to help harmlessly release negative moods or aggression.
Violent reward	Whether game violence provides positive or thrilling emotions such as satisfaction or power.
Social interaction	Playing games with a group; developing personal relationships with other players.
Escapism	Using games to regulate dysphoric moods or to escape the frustrations of daily life.
Loss-aversion	Tendency of a loss to frustrate or to “spoil the fun.” Likely subsumes search for challenge.
Customization	Interest in in-game creative pursuits like personalizing an in-game avatar or building a house.
Grinding/completion	Attitudes toward performing repetitive actions or paying real-life money to earn in-game rewards; interest in performing every possible action in a game or collecting every in-game item.
Autonomy/exploration	Enthusiasm for games with many choices, options, multiple solutions to puzzles, and open areas to explore.

**Table 2:** **List of items in the gaming attitudes, motivations, and experiences scales (GAMES)**.

**STORY**	
1.	Video game stories aren't important to me. [R]
2.	Stories in video games just get in the way. [R]
3.	In video games, it's hard for me to identify with my character. [R]
4.	It's hard for me to play a game if I can't relate to my character.
5.	The feeling of immersion is important to me. (Immersion is feeling like you are really there.)
6.	I really do my best to put myself into the main character's shoes.
7.	I'm interested in learning the lore or history of video game worlds.
8.	I love to learn about the backstories of the characters in video games.
9.	I feel emotionally attached to the characters in my favorite games.
10.	Some of my favorite stories are in video games.
11.	I mostly play video games for their stories.
12.	I'm excited to find out what happens next in the story.
**VIOLENCE CATHARSIS**	
13.	I play violent games to act out my anger without really hurting anyone.
14.	I play violent games when I'm angry or upset to avoid arguing with people I know.
15.	Violent games allow me to release negative energy.
16.	Being violent in a game helps me “get it out of my system.”
17.	Playing violent video games helps me be less violent in real life.
18.	I express my anger in violent video games so I don't act angry in real life.
19.	Video game violence makes me feel better when I'm frustrated.
**VIOLENT REWARD**	
20.	Killing things in the game makes me feel powerful.
21.	Sometimes I'll hack up or shoot enemy corpses, just for fun.
22.	I like violence in my video games - the more violent the better.
23.	Video game violence is exciting and rewarding.
24.	Shooting someone in the head in a game is deeply satisfying.
25.	It feels good to shoot or slice parts off of enemies. (e.g., shooting a head off, or cutting an arm off.)
**SOCIAL INTERACTION**	
26.	When I play video games, I don't feel connected to the other players. [R]
27.	I make more friends by playing video games.
28.	My friends and I use video games as a reason to get together.
29.	Often, a group of friends and I will spend time playing video games.
30.	I enjoy playing video games with a group of my buddies.
31.	I like playing with a group, online or in the same room.
**ESCAPISM**	
32.	Games calm me down when I'm feeling nervous.
33.	I like to do something that I could not normally do in real life through a video game.
34.	I play video games because they let me do things I can't do in real life.
35.	I play video games to keep my mind off my problems.
36.	I play video games because it allows me to escape real life.
37.	Video games allow me to escape from the problems associated with everyday life.
**LOSS-AVERSION**	
38.	Even when I lose, I still have fun. [R]
39.	If I could, I would only play games against weaker players, so I could win more often.
40.	It makes me mad if there are consequences when I mess up in a game, like losing points or getting a bad ending.
41.	I get upset when I lose to other players.
42.	Losing a game always makes me mad - what a waste of time!
43.	Winning is fun; losing isn't.
44.	Losing is frustrating and detracts from my experience.
**CUSTOMIZATION**	
45.	I like making things in video game, like houses or outfits.
46.	I'll put considerable time into designing my character's appearance (e.g., clothes, face).
47.	I like to personalize and customize my character.
48.	I really like to customize my character's outfit.
**GRINDING/COMPLETION**	
49.	I rarely complete collections of in-game items. [R]
50.	I don't mind grinding for an hour or two to get an item I want. (Grinding is doing the same thing over and over).
51.	I'm excited to unlock achievements or earn trophies in games.
52.	I will often level up my characters until they reach the level cap (i.e. they can't level up any further).
53.	I'll play a game until I get a 100% on it, completing everything in the game.
54.	I like taking the time to pick up every single collectible item in the game.
**AUTONOMY/EXPLORATION**	
55.	I like games that offer different ways to get to the next level or area.
56.	I like having a choice of several different places or levels to try.
57.	I like games that offer you a lot of options and choices.
58.	I like games that do not put a lot of constraints on the player.
59.	I prefer games that allow me to play however I want.

*Items marked with [R] are reverse-scored. Items 28, 29, 33, 34, and 41 are adapted from Sherry et al. (2006)*.

**Table 3:** **Factor loadings**.

**Item**	**Story**	**Catharsis**	**Violence**	**Social**	**Escapism**	**Losing**	**Custom**	**Grinding**	**Autonomy**
1	**−0.95**	0.01	−0.02	0.00	0.14	−0.05	0.00	0.07	0.02
2	**−0.92**	0.03	0.03	−0.02	0.09	0.03	0.01	0.12	0.15
3	**−0.51**	−0.01	0.06	0.05	0.09	0.02	−0.10	0.01	0.03
4	**0.46**	0.13	−0.11	−0.06	0.14	0.15	0.11	−0.04	0.05
5	**0.60**	−0.07	0.12	−0.06	0.12	0.05	−0.04	−0.03	0.15
6	**0.67**	0.13	−0.02	−0.06	0.07	−0.04	0.08	0.00	−0.02
7	**0.68**	−0.12	0.15	−0.02	−0.03	−0.11	−0.05	0.05	0.17
8	**0.71**	−0.05	0.07	0.04	−0.03	−0.07	−0.04	0.13	0.15
9	**0.72**	−0.05	0.01	−0.02	0.17	−0.05	0.11	−0.10	−0.07
10	**0.74**	−0.01	0.03	0.07	0.00	0.00	−0.07	0.13	−0.05
11	**0.82**	0.12	−0.15	−0.08	−0.06	0.14	−0.09	0.03	−0.04
12	**0.84**	0.05	0.00	−0.04	−0.05	0.04	−0.05	−0.01	0.00
13	−0.05	**0.68**	0.05	−0.11	0.09	−0.03	−0.02	0.09	0.01
14	−0.03	**0.68**	0.05	0.01	0.01	0.09	−0.01	0.04	0.09
15	0.01	**0.71**	0.02	0.07	0.12	−0.01	0.00	−0.05	−0.01
16	−0.01	**0.74**	0.12	0.00	0.02	0.05	−0.06	−0.01	0.06
17	0.03	**0.88**	−0.06	−0.01	−0.05	−0.06	0.04	−0.06	−0.01
18	0.03	**0.94**	−0.11	−0.01	−0.04	0.01	0.00	−0.01	0.00
19	−0.05	**0.60**	0.29	−0.05	0.09	−0.15	0.03	0.00	−0.13
20	0.02	0.03	**0.54**	0.14	0.18	0.12	0.03	−0.06	−0.06
21	−0.10	−0.06	**0.69**	0.06	−0.03	0.03	0.06	−0.01	0.00
22	0.04	0.15	**0.71**	−0.04	−0.16	0.01	0.02	0.02	0.06
23	0.02	0.09	**0.78**	−0.03	−0.02	0.04	−0.09	0.02	0.02
24	0.04	−0.01	**0.80**	0.01	−0.07	−0.06	0.03	−0.02	−0.04
25	0.00	−0.08	**0.88**	0.01	−0.05	−0.03	0.04	0.03	−0.01
26	−0.14	−0.03	0.08	**−0.54**	0.06	0.04	−0.02	−0.05	0.03
27	0.06	0.19	−0.06	**0.57**	−0.02	0.01	0.02	−0.05	0.09
28	0.13	−0.10	0.07	**0.69**	0.13	0.01	−0.07	0.00	0.02
29	−0.04	−0.03	0.02	**0.83**	−0.01	−0.03	0.02	−0.05	−0.02
30	−0.09	−0.04	0.01	**0.87**	−0.04	0.00	0.01	−0.03	−0.08
31	−0.07	0.05	0.01	**0.80**	−0.22	0.08	−0.07	−0.06	0.09
32	0.14	0.01	0.10	0.14	**0.38**	−0.06	0.10	0.04	−0.16
33	0.02	−0.06	−0.01	0.09	**0.57**	−0.05	−0.10	−0.01	0.05
34	0.05	0.05	−0.11	0.11	**0.58**	0.10	−0.05	−0.03	0.15
35	−0.11	0.06	−0.09	0.01	**0.80**	−0.01	0.06	−0.01	−0.06
36	−0.05	0.02	−0.01	−0.14	**0.83**	0.03	−0.02	0.06	0.02
37	−0.07	0.00	−0.06	−0.08	**0.88**	−0.08	0.03	0.03	−0.04
38	−0.15	0.16	−0.17	0.11	−0.07	**−0.56**	0.04	0.05	0.10
39	−0.10	−0.03	0.10	−0.13	0.03	**0.50**	0.00	0.00	0.01
40	−0.03	0.09	−0.13	0.14	0.01	**0.57**	0.03	0.07	−0.03
41	−0.09	−0.05	0.09	0.07	0.12	**0.58**	0.02	−0.06	0.02
42	0.01	0.15	−0.08	−0.08	−0.07	**0.66**	−0.01	0.00	−0.03
43	−0.06	−0.08	0.02	0.03	−0.02	**0.72**	−0.03	0.03	0.07
44	0.09	−0.03	−0.03	0.04	−0.07	**0.76**	0.05	0.01	0.02
45	0.03	−0.02	−0.03	−0.02	−0.06	−0.04	**0.64**	0.07	0.13
46	−0.03	0.01	0.02	−0.01	0.02	0.02	**0.87**	−0.01	−0.03
47	−0.04	−0.04	0.05	−0.05	0.05	0.01	**0.88**	−0.05	0.09
48	−0.02	0.03	−0.02	0.01	−0.01	0.01	**0.89**	−0.05	−0.02
49	0.00	−0.09	0.05	0.04	0.18	0.01	0.03	**−0.78**	0.05
50	0.02	−0.03	0.02	0.15	−0.02	−0.01	0.16	**0.32**	−0.01
51	0.03	0.02	−0.03	0.06	−0.14	0.21	0.11	**0.40**	−0.10
52	0.03	0.01	0.05	0.07	0.17	−0.06	0.00	**0.60**	−0.02
53	−0.04	−0.03	0.01	−0.06	0.12	0.05	−0.06	**0.80**	0.06
54	−0.06	−0.11	0.04	−0.09	0.09	0.00	−0.03	**0.81**	0.02
55	−0.01	−0.04	0.07	−0.03	0.08	−0.17	−0.07	−0.07	**0.56**
56	−0.01	−0.10	−0.05	0.06	0.06	−0.04	0.09	0.05	**0.64**
57	0.07	0.01	0.03	0.01	−0.07	−0.05	0.02	0.09	**0.64**
58	−0.07	0.02	0.01	−0.04	−0.03	0.12	0.08	−0.13	**0.68**
59	−0.09	0.13	−0.06	−0.04	−0.06	0.06	−0.03	0.06	**0.69**

*Factor loadings of absolute value greater than 0.30 are highlighted in bold*.

**Table 4:** **Inter-factor correlations and Cronbach's alphas**.

	**Story**	**Catharsis**	**Violence**	**Social**	**Escapism**	**Losing**	**Custom**	**Grinding**	**Autonomy**
Story	*0.92*	0.24^***^	0.27^***^	0.51^***^	−0.16^***^	0.31^***^	0.37^***^	0.35^***^	0.34^***^
Catharsis		*0.91*	0.54^***^	0.55^***^	0.22^***^	0.16^***^	0.04	0.24^***^	0.09^**^
Violence			*0.86*	0.48^***^	0.11^**^	0.30^***^	0.07^*^	0.11^**^	0.29^***^
Social				*0.83*	0.06	0.28^***^	0.24^***^	0.26^***^	0.35^***^
Escapism					*0.82*	−0.15^***^	−0.01	0.18^***^	−0.33^***^
Losing						*0.81*	0.13^***^	0.17^***^	0.24^***^
Custom							*0.88*	0.46^***^	0.36^***^
Grinding								*0.79*	0.05
Autonomy									*0.78*

*Cronbach's alphas are displayed on the diagonal in italics*.

* p < 0.05,

** p < 0.01,

*** *p < 0.001*.

### Confirmatory factor analysis

Once a stable EFA solution was found, this EFA-derived factor structure was applied to the second half of the sample (*n* = 332, including 41 undergraduates) using CFA in the “sem” package for R (Fox, 2006). Because responses on items were often non-normal, a maximum likelihood estimation method was deemed inappropriate. Instead, the CFA used generalized least squares (GLS), which relaxes the assumption of multivariate normality.

Results from the CFA demonstrated excellent model fit [X^2^_(1616)_ = 2012, *p* < 0.001, TLI = 0.99, CFI = 0.99, RMSEA = 0.027]. We interpret this well-fitting CFA as evidence for the scales' internal reliability, since the relationships between latent factors and their indicator variables were similar across subsets of participants.

Some participants had responded to all retained items but had been discarded for missing data on other, discarded items. An additional CFA was performed including these participants (*n* = 111, including 21 undergraduates). Model fit remained excellent [X^2^_(1711)_ = 54982, *p* < 0.001, TLI = 0.99, CFI = 0.99, RMSEA = 0.03]. Therefore, these participants were returned to the dataset for all subsequent analyses, increasing the total sample size to *N* = 783.

### Relationships between latent factors and game preferences

#### Game franchises

If the 9-factor solution represents valid individual differences in game preferences, they should covary with specific game franchises. For example, gamers who list story-based franchises (e.g., *Mass Effect*) among their favorites should be higher than average on the Story factor, whereas players who enjoy open-world, free-form games (e.g., *Skyrim*) should have higher-than-average scores on the Autonomy factor. Thus, the following analyses examined whether participants' factor scores could be predicted as a function of the games they had listed among their three favorites.

For each of the 20 most-frequently indicated favorite game franchises, a dummy-coded (0 = no; 1 = yes) vector was created to indicate whether a participant listed that franchise among their top 3 favorite video games. A multiple analysis of variance (MANOVA) was then conducted to determine whether GAMES factor scores could be predicted as a function of the vectors of favorite games. Thus, the analysis compared whether gamers who enjoyed a particular game franchise generally scored lower or higher on a particular factor from the 9-factor solution. All results are presented as Type III Sums of Squares, thereby representing the unique variance in each factor after partialing out the variance due to other game franchises. Analysis was restricted to those participants who had indicated at least one of these 20 game franchises as a favorite (*n* = 531). The influence of each favorite game franchise on each factor is summarized in Table 5.

**Table 5:** **Coefficients of favorite game franchises on GAMES factor scores**.

**Game franchise**	**MANOVA**	***b***								
	***F*_(9, 502)_**	**Story**	**Catharsis**	**Violence**	**Social**	**Escapism**	**Losing**	**Custom**	**Grinding**	**Autonomy**
Zelda	3.72^***^	0.47^***^	−0.12	−0.11	0.20^†^	0.22^†^	−0.18	0.09	0.20^†^	−0.02
Final Fantasy	4.63^***^	0.53^***^	−0.25^†^	0.01	−0.07	0.33^*^	0.14	0.34^*^	0.33^*^	−0.01
Half-Life	2.18^*^	0.37^**^	0.04	0.07	0.14	0.17	0.13	−0.15	−0.19	−0.00
Mass Effect	4.21^***^	0.60^***^	0.14	−0.13	0.00	0.20	0.06	0.21	−0.06	0.21
Fallout 1 & 2	3.22^***^	−0.04	0.01	0.23	−0.46^**^	0.17	0.03	−0.24	−0.19	0.35^*^
Deus Ex 1	4.52^***^	−0.39^**^	−0.27^†^	0.04	0.07	−0.24^†^	−0.18	−0.41^**^	−0.56^***^	0.36^**^
Super Mario	2.36^*^	−0.27^*^	0.08	−0.10	−0.22	−0.15	0.53^***^	−0.02	−0.02	−0.20
Portal	2.63^**^	0.39^**^	0.11	−0.06	0.12	0.19	−0.03	−0.01	−0.19	−0.26
Skyrim	3.91^***^	0.55^***^	0.08	0.15	0.14	0.06	−0.02	0.61^***^	0.57^***^	0.48^***^
Halo	2.13^*^	0.32^*^	−0.02	0.25^†^	0.26^†^	0.09	0.27^†^	−0.07	0.15	0.02
Planescape	3.26^***^	0.36^*^	−0.06	−0.12	−0.37^*^	0.00	−0.19	−0.28	−0.19	0.19
Pokemon	2.20^*^	0.51^***^	0.04	0.17	0.14	0.12	0.28^†^	0.15	0.13	0.03
Call of Duty	3.83^***^	−0.59^***^	0.08	−0.02	0.11	−0.36^*^	0.61^***^	−0.41^*^	−0.08	−0.38^*^
Morrowind	1.50	0.14	−0.26	0.08	−0.19	−0.01	−0.18	0.14	−0.19	0.37^*^
Team Fortress 2	1.02	−0.09	0.05	−0.07	0.22	−0.05	0.19	−0.07	−0.27	−0.10
Minecraft	1.90^*^	0.42^**^	0.01	−0.09	0.39^*^	0.09	−0.08	0.08	0.24	0.31^†^
Grand Theft Auto	2.10^*^	−0.01	0.50^*^	0.63^***^	−0.06	0.24	0.33^†^	0.08	−0.21	0.24
World of Warcraft	2.06^*^	0.25	0.53^**^	0.58^**^	0.29	0.47^**^	0.42^*^	0.01	0.33^†^	0.14
Baldur's Gate	1.79^†^	0.30^†^	−0.33^†^	−0.25	0.00	−0.02	−0.57^**^	0.19	−0.08	0.23
Bioshock	1.96^*^	0.46^**^	0.11	0.20	0.26	0.39^*^	0.02	−0.07	0.08	−0.18

* p < 0.05,

** p < 0.01,

*** p < 0.001,

† *p < 0.10*.

#### Game platforms

Similarly, if the GAMES factors are externally valid, they should be related to players' choices of gaming platforms. For example, serious players who buy hardware specifically to play games should be different from players who only play games incidentally (i.e., on a cell phone or Facebook account owned for primarily non-game reasons). Thus, players who indicated typically using certain platforms may be higher or lower on certain factors than other players. As before, dummy-codes were created for each subject for the platforms they reported using to play games. We performed another MANOVA to see whether choice of platform predicted the 9 factor scores. Age was entered as a covariate. Results are summarized in Table 6. In general, players of dedicated game platforms such as the PC, PS3, and XBOX 360 are higher on Story, Violent Reward, Escapism, and Social Interaction, while players on incidental platforms like phones and Facebook are higher on Loss-Aversion and Grinding.

**Table 6:** **Coefficients of game platform effects on GAMES factors**.

**Platform**	**Story**	**Catharsis**	**Violence**	**Social**	**Escapism**	**Losing**	**Custom**	**Grinding**	**Autonomy**
PC	0.63^***^	0.20^†^	0.64^***^	0.57^***^	0.60^***^	−0.54^***^	−0.04	−0.08	0.60^***^
Wii	−0.06	−0.05	−0.19^**^	−0.12	−0.03	0.12	0.03	0.07	−0.14^†^
PS3	0.33^***^	0.27^**^	0.14^†^	0.14^†^	0.25^**^	0.01	0.09	0.14^†^	0.05
X360	0.32^***^	0.13^†^	0.41^***^	0.40^***^	0.21^**^	0.01	0.10	0.20^**^	0.12^†^
DS/3DS	0.29^**^	−0.04	−0.06	0.10	0.14	−0.00	0.15	0.22^**^	−0.14^†^
PSP	0.03	0.07	0.10	−0.20^†^	−0.03	−0.13	0.14	−0.05	−0.05
Phone	−0.07	−0.00	−0.10	−0.17^*^	−0.11^†^	0.19^*^	0.09	0.15^**^	−0.09
Facebook	−0.21	0.05	−0.07	0.01	−0.05	0.32^*^	0.18	0.13	0.01
Tabletop	−0.16^*^	−0.15^†^	−0.24^***^	0.06	−0.12	−0.14^†^	−0.08	−0.14^†^	−0.22^**^
P&P	0.47^***^	0.31^**^	0.14^†^	0.34^***^	0.20^*^	−0.17^†^	0.28^**^	0.01	0.39^***^
Sports	−0.17^*^	−0.14	−0.27^**^	−0.01	−0.33^***^	0.12	−0.19^*^	0.13	−0.22^**^
Arcade	−0.11	0.05	0.15	0.39^*^	0.02	−0.14	0.22	0.27^†^	0.18
Age	−0.01	−0.02^**^	−0.01	−0.04^***^	−0.01	−0.01	−0.01^*^	−0.02^**^	0.01

*“Tabletop” refers to traditional tabletop board and card games like Euchre, Chess, or Settlers of Catan. “P&P” refers to “pen and paper” roleplaying games such as Dungeons and Dragons, GURPs, or Dogs in the Vineyard. “Sports” refers to traditional real-life sports rather than videogames about these sports. Age is included as a covariate, and its regression coefficients are not standardized*.

*** p < 0.001,

** p < 0.01,

* p < 0.05,

† *p < 0.10*.

#### Patterns of use

A multiple regression was conducted to determine whether factor scores predicted participants' frequency of use, proportion of spare time spent on game use, and self-described attitude toward games (casual or hardcore). Results are summarized in Table 7. In general, higher Story, Violent Reward, Escapism, Social Interaction, and Autonomy scores were associated with playing more frequently, spending a greater proportion of spare time on video games, and self-description as a “hardcore” player. Higher scores on Loss-aversion and Customization were associated with reduced frequency of play, a smaller proportion of spare time spent on games, and self-description as a “casual” player.

**Table 7:** **Relationships between GAMES factors and patterns of game use**.

	**Proportion of spare time**	**Frequency**	**Casualness**
Story	0.23^***^	−0.29^***^	−0.13^**^
Catharsis	−0.08^†^	0.07	0.09^†^
Violence	0.18^***^	−0.25^***^	−0.27^***^
Social	0.12^***^	−0.16^***^	−0.09^*^
Escapism	0.24^***^	−0.16^**^	−0.16^**^
Losing	−0.16^***^	0.17^***^	0.29^***^
Custom	−0.08^*^	0.09^*^	0.17^***^
Grinding	0.03	0.03	−0.04
Autonomy	0.08^†^	−0.13^**^	−0.16^***^
Adjusted R	0.328	0.290	0.259

*Proportion ranged from 1 (Almost none of my spare time) to 5 (Almost all of my spare time). Frequency ranged from 1 (Never) to 7 (Daily). Casualness ranged from 1 (Very hardcore) to 5 (Very casual)*.

* p < 0.05,

** p < 0.01,

*** p < 0.001,

† *p < 0.10*.

#### Correlations with age

Correlations between age and the GAMES factors were inspected via Fisher r-to-z transformations. Forty-seven participants did not give their age and were excluded from this analysis, leaving a sample of *n* = 736; thus, all *t*s represent 734 degrees of freedom. Age was significantly correlated with Catharsis (*r* = −0.08, *p* = 0.004), Loss-Aversion (*r* = −0.10, *p* = 0.001), Social Interaction (*r* = −0.17, *p* < 0.001), Customization (*r* = −0.06, *p* = 0.02), Grinding (*r* = −0.12, *p* < 0.001), and Autonomy (*r* = 0.10, *p* = 0.002). Age was not significantly correlated with Story (*r* = 0.02, *p* = 0.14), Violent Reward (*r* = 0.02, *p* = 0.15), or Escapism (*r* = 0.02, *p* = 0.17).

### Pathology

Items in the pathology questionnaire were scored as 1 for a “Yes” response, as 0 for a “No” or “Not Applicable,” and as 0.5 for a “Sometimes” response, per the recommendations of Gentile et al., (under review). The item “Have you played video games as a way of escaping from problems or bad feelings?” was discarded based on the results from an item response theory analysis of these items (Gentile et al., under review). Items were summed to create a total pathology score for each participant. As recommended by Gentile et al., (under review), the study followed DSM-IV criteria for other disorders and assigned a positive diagnosis to participants who endorsed at least half (7) of the symptoms. The percentage of pathological gamers in the final data was found to be 8.16%, comparable to most similar studies (for a review, see Kuss and Griffiths, 2012). Internet gaming forum members indicated significantly more symptoms than did college undergraduates [*M*s = 3.47 and 2.39, Welch's *t*_(145)_ = 4.64, *p* < 0.001] but were not more likely to reach the threshold for diagnosis (9.09% and 7.14% pathological in the internet and undergraduate samples, respectively, *G* = 0.01, 1 d.f., *p* = 0.92).

To determine whether any of the 9 factors were associated with an increase in the odds of exhibiting game pathology, we conducted a multiple logistic regression, using the factors to predict the probability of a positive diagnosis of pathological game use. Players higher on the Escapism scale were much more likely to have a positive diagnosis of pathological game use than individuals lower on this factor (*OR* = 2.85, *p* < 0.001). Additionally, Social Interaction (*OR* = 1.57, *p* = 0.013) and Grinding (*OR* = 1.49, *p* = 0.029) scores were also significantly associated with increased risk.

A separate multiple logistic regression was conducted to determine whether participants' reported frequency of play, seriousness about games (i.e., “casual” or “hardcore”), and proportion of free time spent on games were associated with the incidence of pathology. Of these, only proportion of free time spent on games was significantly related to pathology (*OR* = 1.97, *p* < 0.001).

## Discussion

The aims of the current report were to develop and validate a measure to assess individual differences in game motives and preferences and to evaluate the extent to which these factors are related to pathological gaming. Based on the EFA and CFA and the analyses including game franchises, this measure appears to demonstrate excellent internal reliability, as evidenced by model fit of the CFA from the split-half analysis, and validity, as evidenced by how game franchise preferences related to the factor structure. Also, while self-selection processes cause the sample to consist of primarily “hardcore” players (White males who play daily), the additional recruitment of 300 college undergraduates helps to diversify the study sample to females and less serious players.

This measure improves on previous instruments in a number of ways. First, it builds upon the latent variables of these previous studies by adding new factors, particularly Story, which has become an increasingly important facet to players in the last decade. We also believe that the Grinding factor is of theoretical importance, and may (in combination with Losing) predict how different players differently satisfy SDT needs for competence. It has been said that there are “two kinds of games: those that are won because of skill, and those that are won because of time” (Baron, 1999). These two factors may predict whether a player will be more likely to find competence satisfaction through blistering challenge or through patient earning of rewards. Additionally, previous studies drew upon limited samples: Yee (2006a,b); Yee et al. (2012) used only players of MMORPGS (a popular, although niche, genre of video games), and Sherry et al. (2006) used only volunteers 23 and younger. Our sample features players from a broad range of ages and genres, which includes both MMORPG players and undergraduates, but additionally many more.

Our factors demonstrated excellent reliability. Moreover, factor scores were found to be related to participants' favorite franchises in sensible ways. For example, fans of role-playing and story-based franchises like *Final Fantasy, Mass Effect, Planescape: Torment*, and *Half-Life* had greater Story scores than did fans of other games. Similarly, players of free-form RPGs like *Skyrim* or *Fallout* had higher Autonomy scores, while players of the carefully-scripted *Call of Duty* franchise had lower Autonomy scores. The 60-or-more-hours-long RPGs *Skyrim* and *Final Fantasy* were each associated with higher Grinding scores. The violent *Grand Theft Auto* was associated with higher Violence Reward and Violent Catharsis.

Our factors also seem to represent differences between players of different game platforms. For example, users of the three most conventional video game platforms—Playstation 3, XBOX 360, and personal computers—placed greater value on video game stories, violence, and escapism. However, PC gamers were also noticeably higher in Autonomy, possibly reflecting this platform's tendency for more open-ended, choice-rich, and modifiable video games. They were also much more capable of tolerating losses. By comparison, players of incidental platforms such as phone and Facebook games found losses to be more frustrating. Phone gamers also scored higher on Grinding. Many phone games involve simple, rapid gameplay with progressively earned in-game currency which can then be traded in for various upgrades (i.e., *Jetpack Joyride, Tiny Tower, Off the Leash*, *Punch Quest*). Moreover, these games are often “free-to-play,” costing nothing to install and instead being funded by players who convert real-world money to in-game currency in order to purchase these upgrades. Since our Grinding scale measures both attitudes toward earning and paying for in-game rewards, we consider this further evidence for the validity of our scales. However, no phone or Facebook games made it into the 20 favorite titles, so it is yet to be determined whether this business model is the actual cause of the observed relationship between phone games and Grinding.

The relationships between certain factor scores and scores on the pathology questionnaire suggest a possible role for this instrument in identifying those at risk for video game overuse. By understanding the motives, habits, and preferred genres of those with video game problems, we can be better equipped to diagnose and treat excessive video game use. For example, players who are trying to “escape” themselves through fantasy immersion or role-play seem to be at enhanced risk. It seems likely that using video games to escape problems may lead to a vicious cycle. It also suggests that pathological game use may be a symptom of other underlying problems (e.g., depression, social phobia) that may be more difficult to treat—if someone is using video games to escape these problems, then abstention from video games might only treat those symptoms of video game use while leaving the underlying problem intact. This replicates previous reports of a link between escapism and pathology from Yee (2006b). This relationship is interesting to note in light of considerations to no longer list “playing to remove dysphoric mood” as a symptom of game pathology, as it appears to be a “socially normative” form of game use (Gentile et al., under review). While escapism may not be a symptom of pathological video game use, it seems to be consistently associated with game pathology (Yee et al., 2012; Kneer and Glock, 2013). We suggest that future research not disregard possible links among dysphoric mood, coping, self-escape, and video game pathology.

We also found some evidence for a player-game interaction in pathology. Players who have higher social motives for playing games are also more likely to have video game pathology. As mentioned in the introduction, games with multiplayer game mechanics and player-to-player relationships may be difficult to quit, since peer pressure and social obligation contribute to continued play. A relationship was also discovered between Grinding and pathology, supporting our hypothesis that those players compelled to grind for hours and complete 100% of the content of their video games would experience greater problems. In previous research, Yee (2006b) suggested a relationship between Advancement motives and Young's diagnostic questionnaire of pathology. The current study replicates this relationship in a broader sample (i.e., players of all games, not just MMORPGs) with a novel measure.

The evidence that problem video game use is related to emotion regulation or self-escape suggests that pathological game use may be motivated by psychological mechanisms similar to those compelling substance abuse (Cooper et al., 1988, 1992, 1995). The prospect that pathological video game use shares an underlying motivation with drug and gambling addictions is theoretically appealing and suggests a reliable and invariable structure underlying addictions in general (Shaffer et al., 2004). However, much more research is needed to verify this possibility.

While MMORPGs such as *World of Warcraft* have long been suspected of being particularly dangerous, the present research provides some insight into why these games may be especially likely to foster problematic use. These games offer all three of the discovered risk factors: Escapism through fantasy immersion; Grinding by earning rewards through frequent play or buying in-game currency with real-world money; and Social Interaction through organized player cooperation, competition, and socialization. Still, we urge researchers of game pathology to consider all manner of games in their research. After all, it was just 30 years ago that “game addiction” was synonymous with single-player arcade action games with no persistent rewards to earn (e.g., *Missile Command, Asteroids, Galaga*). Games in this style are very different from today's MMORPGs. This study provides initial tools to understand game use across many different genres and styles—even sports and other non-video games.

The present research also helps to create a tool for understanding individual differences between players and sources of satisfaction. It has been demonstrated that poor-quality games (bad critical reviews) are worse at satisfying SDT needs than are high-quality games (good critical reviews) (Ryan et al., 2006). However, even between two critically acclaimed games, different players derived different amounts of SDT fulfillment, and enjoyed these games to different degrees accordingly. These motives may interact with properties of a given game to determine how it differentially satisfies psychological needs across different players. For example, players high in Loss Aversion may find a challenging game “frustrating” or “unfair,” while a player with low Loss Aversion may find it thrilling. An understanding of these individual differences may make it easier for game developers, critics, and consumers to understand whether a particular game will suit the consumer's taste. Future research is necessary to demonstrate whether motives and preferences measures can predict player satisfaction.

We would also like to continue developing new items for this scale. In particular, we are uncertain that Loss Aversion, Autonomy, and Customization fully measure the intended constructs. We'd hoped that Loss Aversion would better encompass the whole of competition and challenge, rather than specifically the experience of losing. It is possible that all players enjoy a challenge, so far as it is appropriate to their skill level. Items such as “I find easy games to be too boring” and “I feel proud when I master an aspect of a game” failed to load. Similarly, the Autonomy factor seems to primarily represent the importance of open-world exploration and the diversity of available choices. We had hoped that this factor would also measure the ability to make decisions, explore solutions, and try strategies without intrusive tutorial messages or condescending hints. However, items like “I prefer games that tell you what to do and when to do it” and “I like to figure out games on my own” had pronounced ceiling/floor effects and offered very little variance, thereby failing to load upon any factor. Finally, Customization was not significantly higher for fans of *Minecraft*, perhaps because three of the four items relate to avatar customization and only one item pertains to building things. Future efforts may be able to broaden the scope of this factor.

Additionally, while we achieved acceptable results examining relationships between factor scores and participants' favorite games, future studies could improve upon this approach. First, instructing participants to report three of their “favorite games” induced a certain contamination of nostalgia. Many participants replied with which video games they were playing 10 years ago, rather than which games they would find most enjoyable to play at the given moment. Additionally, the open-response structure of this item did not yield excellent statistical power, as respondents mentioned hundreds of different video games, causing many responses to be discarded and others to be aggregated as best as possible according to the researcher's best judgment. In the future, we plan to constrain choices of favorite games to a robust, diverse, but limited selection of choices.

The current study is limited by its cross-sectional design, which leaves it impossible to determine the direction of causality, if any, in the relationships between motives and pathology. Future longitudinal research is necessary to determine patterns of motive development and pathology over time. Longitudinal data would allow for inspections of Granger causality (Granger, 1969) between motives and pathological status, determining whether motives lead to pathology or pathology leads to motives. Additionally, it would allow us to determine the nature of normative changes in motives over time. The present research cannot disentangle changes in motives due to age from motives associated with age cohort.

This study experienced sharp subject attrition, as many subjects who began the study quit before finishing the survey or made responses of “Not Applicable.” The survey was quite burdensome, taking most participants 20 min or longer to complete. Future research will attempt to use smaller, less burdensome surveys. This will be assisted by the current study, which reduced the GAMES measure from 121 items to 60 items (including the proxy for attention). The smaller pool of items will decrease the time required to complete the survey and the likelihood that at least one question will be marked “Not Applicable,” thereby reducing attrition.

We conclude by urging researchers to consider specific characteristics of players, their personalities, and the games that they play. A common pitfall in video game research is to treat games as being homogenous machines which convert time into virtual gold and slain dragons, or worse, a vehicle for the delivery of scenes of violence to a passive spectator. Players are active participants in their games and exhibit heterogeneous preferences in the games they play. Players are motivated to play games insofar as those games can provide the fulfillment of psychological needs (Przybylski et al., 2010), but different players will seek to fulfill those needs through different ways. To best understand players, preferences, and pathology, we must investigate the interaction of diverse player personality and game mechanisms.

## Author note

This research was supported by a Bond Life Sciences Fellowship awarded to Joseph Hilgard.

### Conflict of interest statement

The authors declare that the research was conducted in the absence of any commercial or financial relationships that could be construed as a potential conflict of interest.

## Footnotes

^1^This adjustment changed neither the number of extracted factors, nor the factor loadings, nor the list of items retained after EFA. This adjustment slightly improved the fit indices of the CFA. Without this adjustment, fit indices were slightly poorer but still quite good [*X*^2^_(1616)_ = 2260.7, *p* < 0.001, *TLI* = 0.99, *CFI* = 0.99, *RMSEA* = 0.030].
